# Depletion of Dendritic Cells Enhances Innate Anti-Bacterial Host Defense through Modulation of Phagocyte Homeostasis

**DOI:** 10.1371/journal.ppat.1002552

**Published:** 2012-02-23

**Authors:** Stella E. Autenrieth, Philipp Warnke, Guido H. Wabnitz, Cecilia Lucero Estrada, Karina A. Pasquevich, Doreen Drechsler, Manina Günter, Kristin Hochweller, Ana Novakovic, Sandra Beer-Hammer, Yvonne Samstag, Günter J. Hämmerling, Natalio Garbi, Ingo B. Autenrieth

**Affiliations:** 1 Interfakultäres Institut für Mikrobiologie und Infektionsmedizin, Universität Tübingen, Tübingen, Germany; 2 Interfakultäres Institut für Zellbiologie, Universität Tübingen, Tübingen, Germany; 3 Institut für Immunologie, Universität Heidelberg, Heidelberg, Germany; 4 Abteilung Molekulare Immunologie, Deutsches Krebsforschungszentrum (DKFZ), Heidelberg, Germany; 5 Abteilung für Pharmakologie und experimentelle Therapie, Institut für experimentelle und klinische Pharmakologie und Toxikologie, Universität Tübingen, Tübingen, Germany; 6 Institut für Molekulare Medizin und Experimentelle Immunologie IMMEI, Universität Bonn, Bonn, Germany; Weill Medical College of Cornell University, United States of America

## Abstract

Dendritic cells (DCs) as professional antigen-presenting cells play an important role in the initiation and modulation of the adaptive immune response. However, their role in the innate immune response against bacterial infections is not completely defined. Here we have analyzed the role of DCs and their impact on the innate anti-bacterial host defense in an experimental infection model of *Yersinia enterocolitica* (Ye). We used CD11c-diphtheria toxin (DT) mice to deplete DCs prior to severe infection with Ye. DC depletion significantly increased animal survival after Ye infection. The bacterial load in the spleen of DC-depleted mice was significantly lower than that of control mice throughout the infection. DC depletion was accompanied by an increase in the serum levels of CXCL1, G-CSF, IL-1α, and CCL2 and an increase in the numbers of splenic phagocytes. Functionally, splenocytes from DC-depleted mice exhibited an increased bacterial killing capacity compared to splenocytes from control mice. Cellular studies further showed that this was due to an increased production of reactive oxygen species (ROS) by neutrophils. Adoptive transfer of neutrophils from DC-depleted mice into control mice prior to Ye infection reduced the bacterial load to the level of Ye-infected DC-depleted mice, suggesting that the increased number of phagocytes with additional ROS production account for the decreased bacterial load. Furthermore, after incubation with serum from DC-depleted mice splenocytes from control mice increased their bacterial killing capacity, most likely due to enhanced ROS production by neutrophils, indicating that serum factors from DC-depleted mice account for this effect. In summary, we could show that DC depletion triggers phagocyte accumulation in the spleen and enhances their anti-bacterial killing capacity upon bacterial infection.

## Author Summary

Dendritic cells (DCs) are professional antigen-presenting cells playing a crucial role in the initiation of T-cell responses to combat infection. DCs adapt their immune response according to the type of pathogen. For example, in response to intracellular bacteria, DCs produce IL-12, thereby initiating Th1 polarization, whereas in response to extracellular parasites or extracellular bacteria, DCs instruct Th2 or Th17 polarization, respectively. Nevertheless, their role in innate immunity is less well understood. To address this, we studied the role of DCs upon infection with the Gram-negative enteropathogenic bacteria *Yersinia enterocolitica* (Ye) and used a mouse model to deplete DCs. We found that DCs have an unexpected role during severe infection as depletion of these cells resulted in better outcome of infection as well as less bacterial load. We also found that DC depletion increased the number of phagocytes with improved anti-bacterial capacity in the spleen. Our study provides new insights into the role of DCs in innate immune response upon bacterial infection and points towards a complex interaction between DCs and phagocyte homeostasis. DC alteration during infection might also be an interesting target for immunotherapy in the future to guide the outcome of infection.

## Introduction

Innate immunity as well as adaptive immunity is involved in the response of the host towards pathogens [1]–[3]. Dendritic cells (DCs) are professional antigen presenting cells playing a central role in immune response by linking the innate and adaptive immunity [4]–[6]. The activation of innate immune cells by microorganisms occurs via binding of pathogen-associated molecular patterns (PAMPs) to pattern-recognition receptors (PRRs), e.g. Toll-like receptors (TLRs) [7]. Upon stimulation by TLR ligands, DCs mature and migrate from the site of infection to secondary lymphoid organs to induce pathogen-specific T-cell responses. Although the role of DCs in the initiation of the adaptive immune response is well established, their impact on immune cells of the innate immune response is less examined.

Previous studies showed that the induction of sepsis in mice resulted in a profound loss of CD11c^+^ DCs from spleen and lymph nodes [8], [9]. The administration of LPS or *Escherichia coli* in mice causes a pronounced reduction in DC numbers in the spleen induced by apoptosis [10], [11]. It was also shown that patients suffering from sepsis displayed increased apoptosis of DCs in the spleen and that an early decrease in circulating DCs was correlated with increased disease severity and mortality [12], [13]. Scumpia et al. showed that DCs were essential in the immune response to sepsis and suggested that strategies to maintain DC numbers or function may improve the outcome during polymicrobial sepsis [14].

We have recently shown that the Gram-negative bacterium *Yersinia enterocolitica* (Ye) affects the homeostasis of the CD4^+^ DCs and, to a lesser extent, the CD8α^+^ DC population in the spleen by the induction of cell proliferation and suppresses *de novo* DC generation [15]. While the role of DCs in adaptive host defense by instructing T cells is well established, their potential contribution to T cell independent innate host defense is poorly understood. In particular, interactions between DCs and phagocytes in the course of infection have not yet been addressed in depth. Therefore, the aim of the study was to address the importance of DCs for the innate immune response *in vivo* upon bacterial infection with Ye. This bacterium causes food borne acute and chronic gastrointestinal and systemic diseases in both humans and mice [16]. By means of its type III secretion system Ye is able to translocate its effector proteins (Yops) directly into the cytosol of host cells [17], thereby preventing its uptake by the target cells.

Phagocytosis and subsequent destruction of the pathogens are critical in the innate immune response. Professional phagocytes, such as neutrophils, macrophages, monocytes and DCs, are specialized to engulf large particles, including microorganisms. Monocytes arise from myeloid progenitors in the bone marrow and are defined as non-dividing circulating blood cells with a half-life of one day in mice [18]. Mouse blood monocytes express CD115, CD11b and low levels of F4/80 and can be distinguished by the expression of Ly6C and CX_3_CR1 into Ly6C^hi^CX_3_CR1^lo^CCR2^+^CD62L^+^ and Ly6C^lo^CX_3_CR1^hi^CCR2^−^CD62L^−^ monocytes [18]. Neutrophils are terminally differentiated effectors and the first cells to migrate toward sites of infection. Release of neutrophils from the bone marrow is mediated by the concerted action of G-CSF, CXCL1, and CXCL2 [19], [20]. At the site of infection, neutrophils engulf and kill bacteria through the production and secretion of proteases, reactive oxygen species and other proinflammatory mediators. Furthermore, neutrophils control the recruitment of other cells (T cells, NK cells, macrophages, and immature DCs) through the production of the chemokines CXCL1, CCL3, and CCL4 [21]. Early upon activation DCs also produce IL-8 thereby attracting neutrophils which leads to colocalization of neutrophils and immature DCs [22]. Mouse neutrophils express TLR2, TLR4 and TLR9 mRNAs [23], and can be activated by LPS leading to shedding of L-selectin and upregulation of CD11b [24].

In this study, we have used an inducible mouse model allowing depletion of CD11c^hi^ DCs by administration of diphtheria toxin (DT) to directly address their impact during initiation of innate immune response upon Ye infection *in vivo* [25]. We found that DC depletion *per se* increased the number of phagocytes and enhanced their anti-bacterial host defense in the spleen leading to increased survival of the mice upon Ye infection.

## Results

To examine the importance of CD11c^hi^ DCs in a bacterial infection model we used a BAC transgenic mouse model to inducibly deplete the DCs. In this model the human diphtheria toxin (DT) receptor (DTR) is expressed under the control of the CD11c promotor allowing depletion of CD11c^hi^MHCII^+^ DCs (∼90% efficacy in the spleen; Figure S1A) by DT administration (CD11c.DOGxC57BL/6 mice herein after referred to as DC-depleted or CD11c.DOG mice, [25]). Repetitive DT administration did not result in reduced survival or weight loss. DT had no effect on the number or percentage of DCs in control mice (C57BL/6 mice; data not shown).

### DC depletion decreases bacterial load and improves survival upon *Yersinia* infection

To address the impact of DCs on the survival of the mice upon a severe bacterial infection we administered DT i.p. one day prior to i.v. infection with 5×10^4^ Ye to DC-depleted and control mice. DT was administered daily during the whole period of observation and survival was analyzed for up to 14 days.

**Figure 1:**
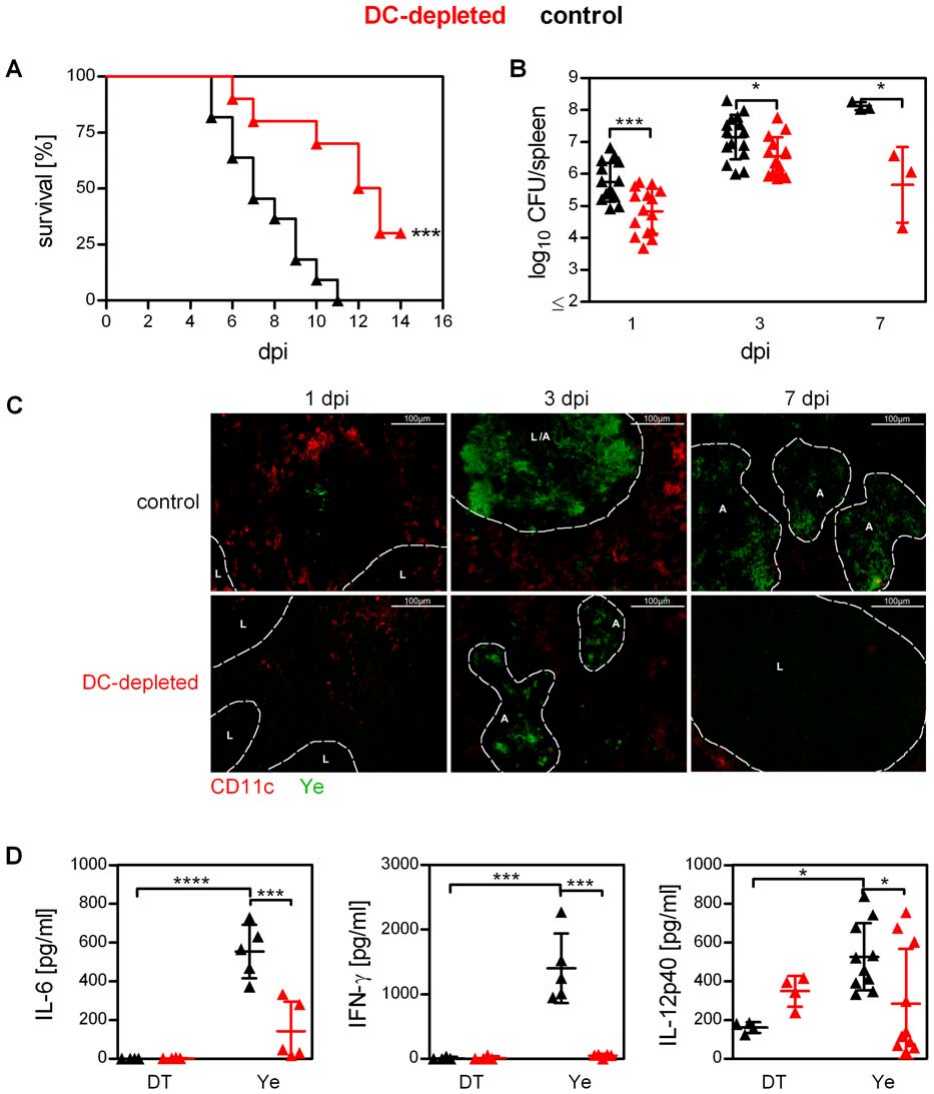
Impact of DC depletion on the outcome of Ye infection. DC-depleted (red symbols) and DT-treated control (black symbols) mice were injected i.v. with 5×10^4^ Ye pYV^+^ (**A–D**) and daily with diphtheria toxin (DT) starting one day before infection. (**A**) Survival mice infected as described above was monitored until day 14 after infection. *** p<0.005 (Log-rank (Mantel-Cox) test). (**B**) Bacterial load (CFU) in the spleen was assessed at the indicated days post infection by plating. Each symbol represents an individual mouse; horizontal lines indicate the mean ± SD. * indicates statistically significant differences (Student's *t*-test). (**C**) Immunohistochemical analysis of Ye (green) and DCs (red) in spleens, visualized by staining with polyclonal antiserum to Ye and Alexa Fluor 488-labeled secondary antibody followed by biotin-labeled monoclonal antibody to CD11c and Alexa Fluor 546-labeled streptavidin. Original magnification ×20; A: abscess, L: lymph follicle. (**D**) 24 h post DT treatment (DT) and 24 h post infection (Ye) serum was collected and concentrations of the indicated proinflammatory cytokines were analyzed by bioplex assay or ELISA. Each symbol represents an individual mouse; horizontal lines indicate the mean ± SD. * indicates statistically significant differences (one-way ANOVA with Bonferroni post test.). Data are from 2 to 4 (**A** and **B** 1 and 3 dpi) or representative of 2 (**C** and **D**) independent experiments.

DC-depleted mice survived significantly longer than control mice with median survival of 12.5 days and 7 days, respectively (p<0.005), indicating that DC depletion was beneficial for the survival upon a lethal Ye infection (Figure 1A). The survival rate correlated with the bacterial load in the spleen which displayed an increase in the colony forming units (CFU) over time (Figure 1B). Overall, we observed significantly less bacterial load in the DC-depleted mice compared to control mice (Figure 1B). Since the cellular composition of the spleens is changing with treatment and infection we analyzed the CFU/g spleen (Figure S1B) observing similar results. CD11c.DOG mice without DT treatment showed similar CFU in the spleen upon Ye infection as control mice (Figure S1C), excluding intrinsic differences in the susceptibility of the mice which were used in this study.

In addition, we performed immunofluorescence microscopy of cryosections from the spleen of Ye-infected mice staining both CD11c^+^ cells and Ye. In agreement with flow cytometry analysis (Figure S1A) the number of CD11c^+^ cells was found to be low in DC-depleted mice due to DT administration. Ye infection led to a decrease in the number of CD11c^+^ cells in control mice (Figure 1C). This is consistent with previous findings from our group [15]. Moreover, massive abscess formation in the spleen of control mice was observed from 3 to 7 days post infection (dpi), whereas in the spleen of DC-depleted mice only small abscesses were found at 3 dpi (Figure 1C). Sepsis is characterized by increased levels of proinflammatory cytokines. One day post Ye infection the levels of the proinflammatory cytokines IL-6, IFN-γ, and IL-12p40 were 2 to 10-fold increased in sera from control mice compared to infected DC-depleted mice or mice without infection, indicating that DCs promote the production of these proinflammatory mediators upon Ye infection (Figure 1D).

Altogether, these studies demonstrate that the depletion of DCs is beneficial for survival upon severe bacterial infection and is associated with lower bacterial load and lower production of proinflammatory cytokines.

### DC depletion leads to replacement of DCs by neutrophils and monocytes

**Figure 2:**
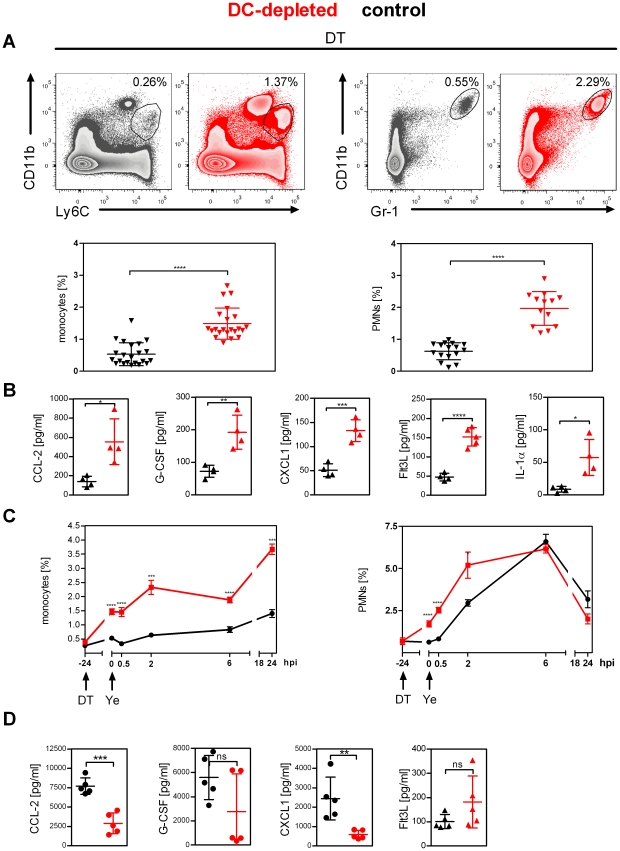
Replacement of DCs by monocytes and neutrophils upon DC depletion. (**A–D**) Mice were treated daily with diphtheria toxin (DT) (**A and B**) starting one day before infection and were injected with 5×10^4^ Ye pYV^+^ for 24 h (**C and D**). (**A**) Representative dot plots showing analysis of monocytes (left) and neutrophils (right) in spleen from control (black) and DC-depleted (red) mice. Numbers adjacent to outlined areas indicate frequency of monocytes and neutrophils. Graphs show the frequency of monocytes (left) and neutrophils (right) per spleen. (**B**) Bioplex assay or ELISA of chemokine concentrations in sera from control and DC-depleted mice 24 h after DT-treatment. (**C**) Flow cytometry analysis of the frequency of monocytes (left) and neutrophils (right) in the spleen at the indicated times post Ye infection. (**D**) Bioplex assay or ELISA of chemokine and cytokine concentrations in sera from control and DC-depleted mice 24 h after Ye infection. Each symbol represents an individual mouse; small horizontal lines indicate the mean ± SD. * indicates statistically significant differences between control and DC-depleted mice (Student's *t*-test). Data are from 5 (**A**) or one representative out of 2 or more (**B**, **C** and **D**) independent experiments.

As DC-depleted mice displayed a significantly lower bacterial load in the spleen, already 1 dpi compared to control mice (Figure 1B) we hypothesized that this could reflect an altered splenocyte composition following DC depletion prior to infection. In fact, single DT treatment of uninfected mice led to a 3 to 4-fold increase in the frequency of inflammatory monocytes (Gr-1^+^Ly6G^−^Ly6C^hi^CD11b^+^) and neutrophils (Gr-1^hi^Ly6G^+^Ly6C^−/int^CD11b^hi^, see Figure S2 for detailed gating strategy) after 24 h in the spleen of DC-depleted mice compared to DT-treated control mice (Figure 2A and [25]). Similarly, increased numbers of neutrophils and monocytes were observed in peripheral blood (data not shown). However, we did not observe differences in the frequency of B cells, T cells or NK cells (data not shown and [25]).

To rule out the possibility that massive DC cell death and/or the phagocytosis of the DC debris might serve as a general pro-inflammatory signal and thereby causing the recruitment of neutrophils, we analyzed neutrophil numbers in the spleen from mixed bone marrow chimeras (80% CD11c.DOG/20% C57BL/6). In these mice, a single DT treatment leads to the depletion of most DCs. However, 10 daily DT applications result in depletion of the pre-existing DCs and those that are continuously being generated from CD11c.DOG progenitors, while the C57BL/6 DC pool expands until reconstituting the whole compartment [26]. Thus, 10 days of DC depletion in these chimeric mice results in a normal DTR^−^ DC compartment with massive DTR^+^ DC depletion. As expected, single DT treatment led to neutrophilia, similar to that in CD11c.DOG mice (Figure S3). Interestingly, 10 days of DT treatment in the chimeric mice revealed only a minor increase in the number of splenic neutrophils (about 2.7-fold) compared to the 27-fold increase observed in CD11c.DOG mice (Figure S3). Similar results were observed when quantifying the frequency of neutrophils (Figure S3). Furthermore, we found that the increased numbers of phagocytes in the spleen upon DC depletion are accompanied by significantly elevated serum levels of CCL2, G-CSF, CXCL1, Flt3L, and IL-1α from DC-depleted mice compared to control mice (Figure 2B), all of which have been shown to be involved in leukocyte recruitment or maintenance [27]–[30]. These data point towards a regulation of neutrophil numbers by DCs via the repression of chemokines/growth factors rather than merely a side effect caused by DC death.

A further increase in the frequency of monocytes and neutrophils in the spleen of both DC-depleted and control mice was observed upon Ye infection. The frequency of monocytes in the spleen of DC-depleted mice continuously increased up to 1 dpi and was 2.5-fold higher compared to control mice (Figure 2C). The frequency of neutrophils continuously increased up to 6 h post infection. This was more pronounced in control mice, and reached similar frequency and numbers 6 h and 1 dpi in both groups of mice (Figure 2C and Figure S4). This is accompanied by significantly elevated serum levels of CCL2, G-CSF, and CXCL1 in control mice 1 dpi compared to DC-depleted mice (Figure 2D). These data suggest different recruitment kinetics of monocytes and neutrophils in response to Ye, similar as shown for intradermal *E. coli* infection [31].

**Figure 3:**
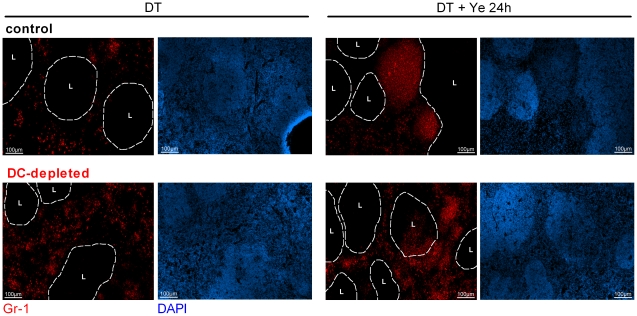
Accumulation of neutrophils in the splenic red pulp upon DC depletion. Immunohistochemical analysis of neutrophils (red) in the spleen 24 h post DT treatment (left) and 24 h Ye infection (right), visualized by staining with biotin-labeled monoclonal antibody to Gr-1 and Alexa Fluor 546-labeled streptavidin. Original magnification ×10. L: lymph follicle. Data are representative of 2 independent experiments. The lymph follicle region is defined by staining nuclei with DAPI (blue).

Immunofluorescence microscopy confirmed the increase in the number of Gr-1^+^ cells in the red pulp upon DC depletion. Ye infection led to increased numbers and accumulation of Gr-1^+^ cells in the splenic red pulp of control mice and was associated with the formation of abscesses, whereas in DC-depleted mice the Ye-induced increase in Gr-1^+^ cells was more uniformly distributed and associated with the formation of microabscesses (Figure 3).

In summary, our data indicate that DCs may regulate the numbers of splenic neutrophils and monocytes associated with increased chemokine production by a yet unknown mechanism.

### DC depletion increases the number of phagocytes with intracellular Ye

**Figure 4:**
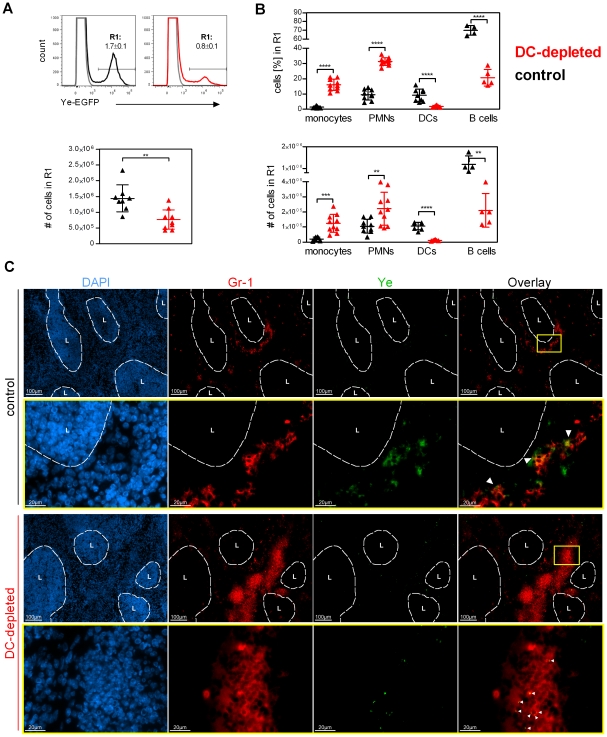
Ye are predominantly associated with phagocytes upon DC depletion. (**A**) Flow cytometry analysis of Ye-GFP^+^ cells in spleens of DT-treated control (black symbols) and DC-depleted (red symbols) mice 30 min after injection of 5×10^8^ GFP-expressing Ye pYV^+^. Numbers adjacent to R1 indicate the frequency of Ye-GFP^+^ splenocytes. Graph shows the total number of Ye-GFP^+^ splenocytes (R1) per spleen. (**B**) Graphs show the frequency and total numbers (#) of Ye-GFP^+^ cells (R1 from A) being monocytes, neutrophils, DCs, and B cells. Each symbol represents an individual mouse; horizontal lines indicate the mean ± SD. * indicates statistically significant differences (Student's *t*-test). (**C**) Immunohistochemical analysis of Ye (green) and neutrophils (red) in the spleen of DT-treated control mice (black) and DC-depleted mice (red) infected as described in (A), visualized by staining with polyclonal antiserum to Ye and Alexa Fluor 488-labeled secondary antibody followed by biotin-labeled monoclonal antibody to Gr-1 and Alexa Fluor 546-labeled streptavidin. The lymph follicle region is defined by staining nuclei with DAPI (blue). Original magnification ×10 (top row) and ×63 (bottom row). L: lymph follicle. Arrows indicate Ye associated with Gr-1^+^ cells. Data are from 2 independent experiments.

As DC depletion led to increased numbers of monocytes and neutrophils in the spleen, both of which are professional phagocytes, we hypothesized that these cells account for lower bacterial load observed already 30 min after Ye infection of DC-depleted mice (Figure S5). Therefore, phagocytosis of eGFP-expressing Ye by splenocytes was analyzed 30 min post intravenous administration. Flow cytometry analysis revealed two times less splenocytes associated with eGFP-Ye (referred to as Ye^+^ cells) (Figure 4A, R1, p<0.001) in the spleen from DC-depleted mice compared to control mice. Detailed flow cytometry analysis showed, however, striking differences in Ye^+^ cells in the various spleen cell subpopulations. In fact, Ye^+^ splenocytes from control mice comprised predominantly B cells (70%) as well as DCs and neutrophils (each 10%), whereas the Ye^+^ splenocytes from DC-depleted mice comprised predominantly neutrophils (32%) and monocytes (17%) (Figure 4B). Calculation of the total numbers of Ye^+^ cells per spleen revealed 2.2×10^5^ Ye^+^ neutrophils, 1.2×10^5^ Ye^+^ monocytes, and 2.0×10^5^ Ye^+^ B cells in DC-depleted mice compared to 1.0×10^5^ Ye^+^ neutrophils, 0.2×10^5^ Ye^+^ monocytes, and 12.0×10^5^ Ye^+^ B cells in control mice (Figure 4B), demonstrating that DC depletion increased the number of phagocytes associated with Ye *in vivo*, whereas the number of Ye^+^ B cells was dramatically reduced.

Furthermore, immunofluorescence microscopy of cryosection from the spleen of DC-depleted mice 30 min post Ye infection revealed a low number of Ye and these were found next to clusters of Gr-1^+^ cells (Figure 4C). In contrast, Ye colonies were obvious in the spleen from control mice and these were partially associated with a lower number of Gr-1^+^ cells as found in DC-depleted mice (Figure 4C). In addition, analyzing the cell contact of neutrophils with DCs by immunofluorescence microscopy, we hardly observed colocalization of Ly6G^+^ cells with CD11c^+^cells in control mice (Figure S6), arguing against a direct cell contact-dependent regulation of neutrophils by DCs.

**Figure 5:**
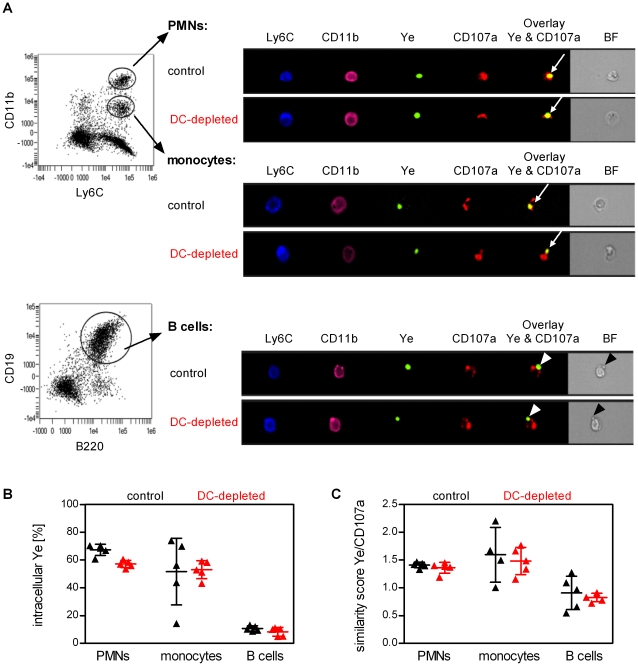
DC depletion does not enhance bacterial uptake. Multispectral imaging flow cytometry analysis of Ye-GFP^+^ cells in splenocytes of DT-treated control (black symbols) and DC-depleted (red symbols) mice 30 min after injection of 5×10^8^ GFP-expressing Ye pYV^+^. (**A**) Dot plots show gating of neutrophils, monocytes, and B cells. Microscopic pictures show representative neutrophils, monocytes, and B cells from DC-depleted or control mice harboring Ye and stained additionally with the lysosomal marker CD107a. Arrows indicate intracellular Ye, arrowheads extracellular Ye. Quantitative analysis of intracellular Ye (**B**, defined as described in Materials and Methods) or similarity score of eGFP-Ye and CD107a (**C**) in neutrophils, monocytes, and B cells from DC-depleted or control mice. Each symbol represents an individual mouse; horizontal lines indicate the mean ± SD. Data are from one out of two independent experiments with similar results.

To dissect whether Ye^+^ splenocytes reflect Ye associated with the membrane of the cells or Ye engulfed by the cells, we used multispectral imaging flow cytometry combining flow cytometry with microscopy at the single cell level. B cells were stained with CD19 and B220 antibodies, whereas monocytes and neutrophils were distinguished by CD11b and Ly6C surface staining (Figure 5 and Figure S2). Intracellular Ye were defined as described in Materials and Methods.

By analyzing phagocytosis with this technique we found that 50–70% of neutrophils and monocytes harbor intracellular Ye. In contrast, only 10% of all Ye associated with B cells were intracellularly located. The frequencies of intracellular Ye in the various spleen cell subpopulations were similar in DC-depleted and control mice (Figures 5A and B), indicating no differences in the phagocytosis rate of the splenocytes.

In addition, colocalization of Ye with CD107a (LAMP-1) protein expressed in late endosomes and lysosomes was analyzed as indicators of bacterial processing. Intracellular Ye in neutrophils and monocytes colocalized with the lysosomal marker CD107a (Figures 5A and C: similarity score Ye/CD107a >1) but no difference was obvious in the phagocytes from DC-depleted and control mice, indicating similar bacterial processing rates in both groups of mice.

Taken together, our data show that DC depletion did not affect the capacity of neutrophils and monocytes to engulf and process Ye *in vivo*. However, DC depletion led to a strong accumulation of neutrophils and monocytes in the spleen resulting in Ye being predominantly associated with these phagocytes.

### Phagocytes from DC-depleted mice are more effective in bacterial killing

Despite similar bacterial phagocytosis and processing rates in DC-depleted and control mice, we hypothesized that the differences in the bacterial load were due to increased intracellular killing mechanism by the phagocytes from DC-depleted mice.

**Figure 6:**
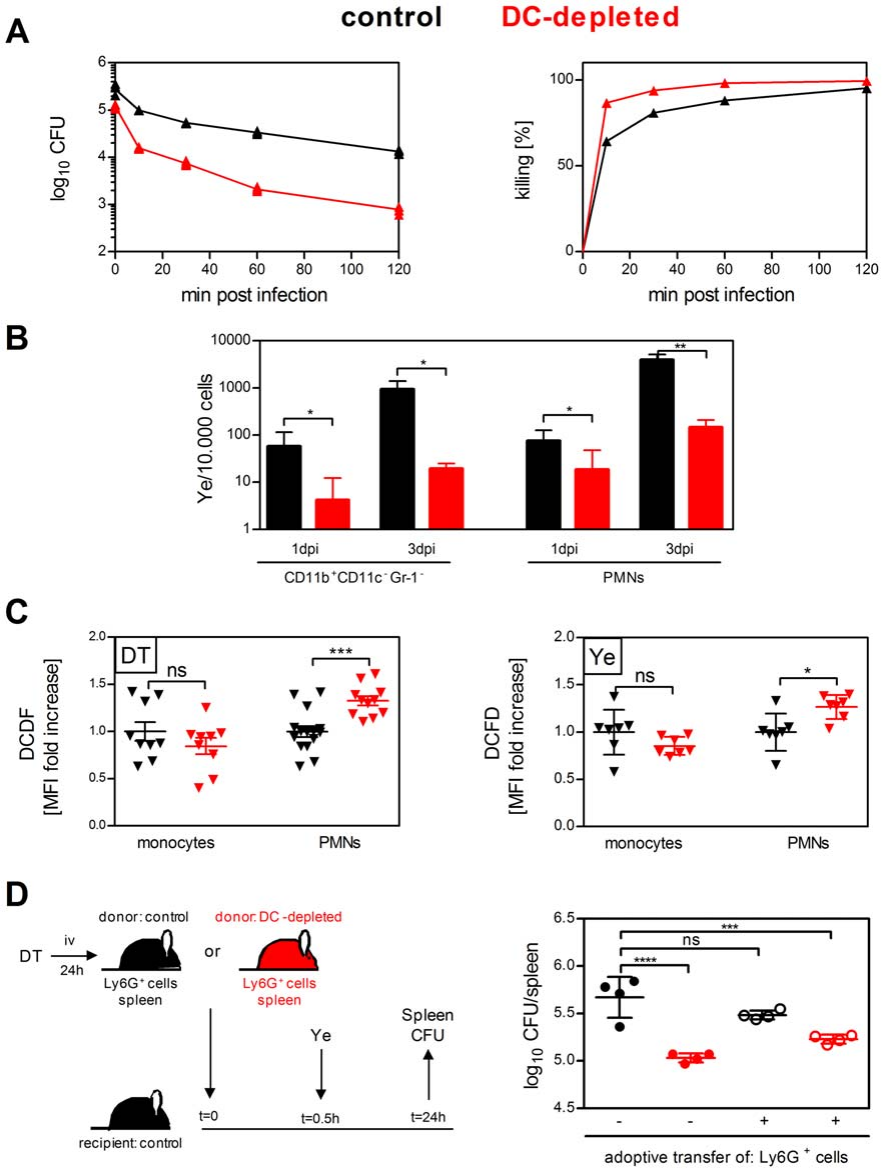
Phagocytes from DC-depleted mice are more effective in bacterial killing. (**A**) Splenocytes from DT-treated control (black symbols) and DC-depleted (red symbols) mice were incubated *in vitro* with Ye (MOI 1) for 10 min. Cells were extensively washed and either plated directly on MH agar plates or incubated further in the presence of gentamicin for the indicated time points before plating. The diagrams show the CFU as log scale (left) and the frequency of killed Ye (right) from one representative out of two experiments with quintuplicates including mean ± SD. (**B**) Viable intracellular *Yersinia* from sorted CD11b^+^Gr-1^−^ spleen cells or neutrophils of DT-treated control (black symbols) and DC-depleted (red symbols) mice injected with 5×10^4^ Ye pYV^+^ were analyzed by plating serial dilutions 1 and 3 dpi. CFU were analyzed per 10.000 sorted spleen cells. Data are from two independent experiments with three to six mice per group (mean ± SD). * indicates statistically significant differences (Student's *t*-test). (**C**) Flow cytometry analyses of ROS production in monocytes and neutrophils from DT-treated control (black symbols) and DC-depleted (red symbols) uninfected mice (left diagram) or injected for 2 h with 5×10^4^ Ye pYV^+^ (right diagram) using 2′, 7′-Dichlorofluorescin diacetate reagent (DCFD). The graph shows fold increase of median fluorescence intensity of DCFD in monocytes and neutrophils from DC-depleted mice compared to control mice. Each symbol represents an individual mouse; horizontal lines indicate the mean ± SD. * indicates statistically significant differences (one-way ANOVA with Bonferroni post test). Data are from 6 independent experiments. (**D**) Splenic neutrophils from DT-treated control mice (black open circles) or DC-depleted mice (red open circles) were purified, adoptively transferred into control mice and infected with 5×10^4^ Ye 30 min later. Control (black circles) or DC-depleted (red circles) mice received PBS instead of neutrophils. The CFU per spleen were analyzed by plating serial dilutions 1 dpi. Each symbol represents an individual mouse; horizontal lines indicate the mean ± SD. Data are from one out of three independent experiments with similar results (three to five mice per group). * indicates statistically significant differences compared to control mice without adoptive transfer (one-way ANOVA with Bonferroni post test).

Performing an *in vitro* killing assay, we observed as early as 10 min after incubation of splenocytes with Ye (multiplicity of infection 1), that the number of recovered intracellular bacteria was reduced by 86.6% in splenocytes from DC-depleted mice compared to only 64.3% in splenocytes from control mice (Figure 6A). These data indicate that DC depletion resulted inmore efficient killing of Ye by splenocytes compared to the killing capacity by splenocytes from control mice.

Additionally, the number of living intracellular Ye in sorted CD11b^+^Gr-1^−^ cells and neutrophils was 4 to 14-fold higher one day and 27 to 49-fold higher three days post Ye infection in control mice compared to DC-depleted mice (Figure 6B), indicating a better bacterial killing by the phagocytes from DC-depleted mice. To corroborate this hypothesis, ROS production was analyzed in both mice without and with Ye infection *in vivo*. We observed increased ROS levels in neutrophils from DC-depleted mice prior to infection (Figure 6C left diagram) as well as 2 h post Ye infection (Figure 6C right diagram), indicating that the neutrophils were activated upon DC depletion and infection. We did not observe differences in ROS production by monocytes neither with nor without infection (Figure 6C). These data provide evidence that DCs not only affect the number of neutrophils in the spleen but also increase their anti-bacterial killing capacity.

To directly demonstrate that the increased number of neutrophils in combination with their increased ROS production in DC-depleted mice account for the initially decreased bacterial load in the spleen, neutrophils from DC-depleted mice were purified and adoptively transferred to control mice. These mice were then infected with Ye (see Figure 6D). As a control, purified neutrophils from control mice were transferred into control mice prior to Ye infection. The bacterial load in the spleen of mice adoptively transferred with neutrophils from DC-depleted mice was significantly reduced compared to control mice without adoptive transfer and similar to that of DC-depleted mice 1 dpi (Figure 6D). In contrast, adoptive transfer of neutrophils from control mice into control mice did not lead to a significant reduction of the bacterial load 1 dpi with Ye (Figure 6D). In summary, our data demonstrate that DC depletion leads to increased number of phago cytes in >the spleen being highly effective in the clearance of bacteria.

### Serum factors from DC-depleted mice increase ROS production by neutrophils and enhance their killing capacity

**Figure 7:**
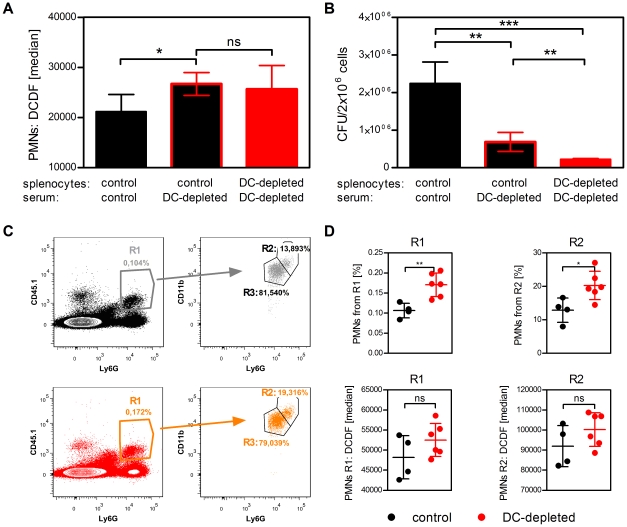
Serum factors from DC-depleted mice increase ROS production by neutrophils and enhance their killing capacity. (**A**) Splenocytes from control or DC-depleted mice were incubated with serum from DT-treated control or DC-depleted mice in medium (1∶5) for 1 h, followed by flow cytometry analyses of ROS production in neutrophils using DCFD. The graph shows the median fluorescence intensity of DCFD in neutrophils. Bars indicate the mean ± SD of triplicates. Data are the combination of 2 independent experiments. * indicates statistically significant differences (one-way ANOVA with Bonferroni post test). (**B**) Splenocytes were incubated with serum as described in (A) and incubated *in vitro* with Ye (MOI 1) for 10 min and plated directly on MH agar plates. The graph shows the CFU of triplicates including mean ± SD. * indicates statistically significant differences (one-way ANOVA with Bonferroni post test). Data are from one out of three independent experiments with similar results. (**C**) Neutrophils from CD45.1 mice were adoptively transferred into control (upper panel) or DC-depleted mice (lower panel). Flow cytometry analysis of transferred CD45.1^+^Ly6G^+^ neutrophils was performed 2 h after transfer. Numbers adjacent to outlined areas indicate frequency of transferred CD45.1^+^Ly6G^+^ neutrophils (R1), CD45.1^+^CD11b^hi^Ly6G^hi^ neutrophils (R2), and CD45.1^+^CD11b^+^Ly6G^+^ neutrophils (R3). (**D**) Frequency of (upper panel) and ROS production by (lower panel) transferred neutrophils (R1 and R2) as described in (C) was analyzed 2 h post neutrophil transfer. Each symbol represents an individual mouse; horizontal lines indicate the mean ± SD. * indicates statistically significant differences (Student's *t*-test). Data are from one out of two (C and D) independent experiments with similar results.

In order to analyze whether serum factors mediate the increased ROS production and killing capacity of neutrophils, we incubated splenocytes from control mice with serum from DC-depleted or control mice for 1 h. The analysis of ROS production by flow cytometry revealed a significant increase in ROS production by neutrophils incubated with serum from DC-depleted mice compared to neutrophils incubated with serum from control mice (Figure 7A). ROS production in neutrophils from control mice incubated with serum from DC-depleted mice was comparable to neutrophils from DC-depleted mice incubated with serum from DC-depleted mice (Figure 7A). Furthermore, splenocytes from control mice showed increased bacterial killing after incubation with serum from DC-depleted mice compared to incubation with control serum (Figure 7B). These data indicate that indeed factors in the serum from DC-depleted mice cause elevated ROS production in neutrophils and enhance the bacterial killing capacity of splenocytes *in vitro*.

To evaluate these findings *in vivo*, purified neutrophils from control mice (CD45.1^+^) were adoptively transferred into either control mice or DC-depleted mice and analyzed for ROS production 2 h after transfer. We observed a higher frequency of transferred CD45.1^+^Ly6G^+^ neutrophils (Figure 7C, R1 and Figure 7D) in the spleen of DC-depleted compared to control mice. This indicates a better attraction of PMNs into the spleen upon DC-depletion. Furthermore, neutrophils tended to produce more ROS when transferred into DC-depleted mice compared to transfer into control mice, although differences in ROS production were statistically not significant (Figure 7D). Within these transferred neutrophils two subpopulations could be distinguished by their expression of CD11b and Ly6G (Figure 7C, R2: CD11b^hi^Ly6G^hi^ and R3: CD11b^+^Ly6G^+^). The frequency of CD11b^hi^Ly6G^hi^ neutrophils was also significantly increased in the spleen of DC-depleted mice compared to control mice (Figure 7C, R2 and Figure 7D). ROS production by these activated transferred CD11b^hi^Ly6G^hi^ neutrophils was twice as high as ROS production by all transferred neutrophils and higher by CD11b^hi^Ly6G^hi^ neutrophils transferred into DC-depleted mice than transferred into control mice.

### DC depletion decreases bacterial load upon bacterial infection

**Figure 8:**
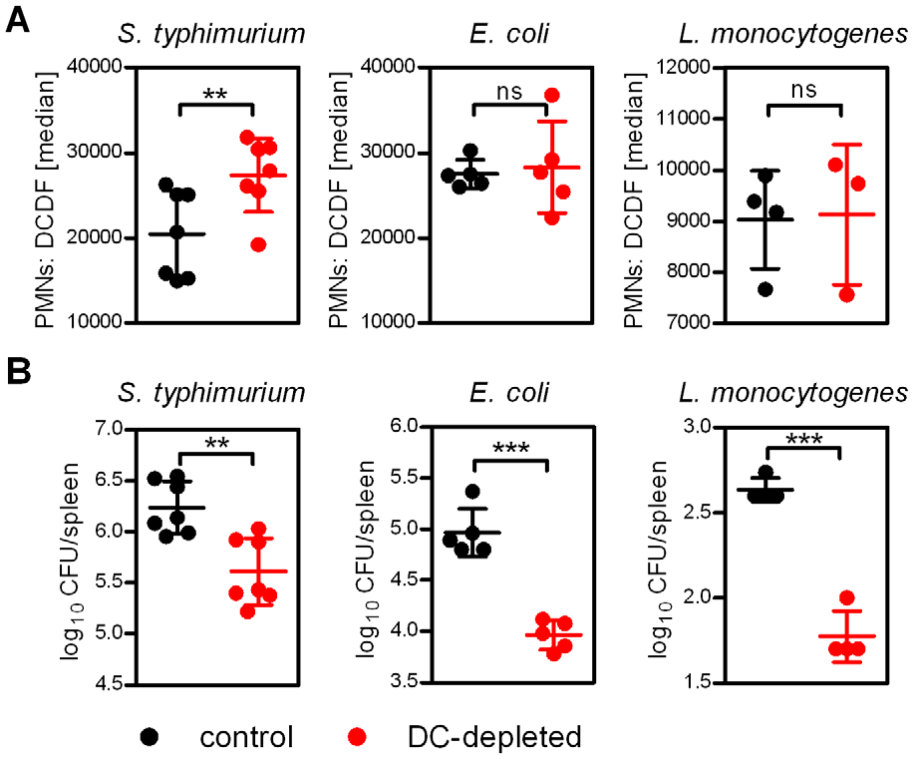
DC depletion decreases bacterial load upon bacterial infection. DC-depleted (red circles) and DT-treated control (black circles) mice were injected with 5×10^4^ *S. typhimurium*, 5×10^7^ *E. coli* or 5×10^4^ *L. monocytogenes* for 2 h. (**A**) Flow cytometry analyses of ROS production in neutrophils 2 h post infection using DCFD. The graphs show the median fluorescence intensity of DCFD. Each symbol represents an individual mouse; horizontal lines indicate the mean ± SD. * indicates statistically significant differences (Student's *t*-test). Data are from 1 or 2 independent experiments. (**B**) Bacterial load (CFU) in the spleen was determined 2 h post infection by plating. Each symbol represents an individual mouse; horizontal lines indicate the mean ± SD. * indicates statistically significant differences (Student's *t*-test). Data are from 1 experiment.

To elucidate whether the increased ROS production by neutrophils in DC-depleted mice and upon bacterial infection as well as their enhanced bacterial killing capacity is specific for Ye or a more general host defense mechanism against other bacteria as well, we analyzed the ROS production by neutrophils and the bacterial load in the spleen 2 h post infection with *Salmonella typhimurium*, *Listeria monocytogenes* and *E. coli*. ROS production by neutrophils was significantly increased in DC-depleted mice compared to control mice upon infection with *S. typhimurium*, but not with *L. monocytogenes* and *E. coli* (Figure 8A). This indicates that different bacteria differently affect ROS production by neutrophils. Nevertheless, the bacterial load in the spleen of DC-depleted mice was significantly reduced compared to control mice upon infection with all three bacteria (Figure 8B), indicating that the increased ROS production by neutrophils prior to infection leads to reduced bacterial load of several pathogens in the spleen. Taken together, our data provide evidence that the increased number of phagocytes combined with the enhanced killing capacity of neutrophils upon DC depletion is at least initially beneficial for the host by reducing the bacterial load upon infection.

## Discussion

The innate immune system is important for pathogen clearance. The role of DCs in the adaptive immune response is well established [4]. However, their function in the innate immune response against bacterial infections is not completely defined. In the present study we used a well established mouse infection model with the extracellular Gram-negative bacterium Ye in DC-depleted mice to define the impact of DCs on the innate immune defense against this pathogen. Ye infection of DC-depleted mice revealed a reduced bacterial load in the spleen compared to infected control mice. We found that DC depletion in these mice led to an increase in the number of neutrophils and monocytes in the spleen one day after DT treatment with a peak at day two after daily DT administration, as recently described [25]. In fact, prior to infection of mice with Ye we observed 1.2×10^6^ more neutrophils and 7×10^5^ more monocytes in the spleen of DC-depleted mice compared to control mice, demonstrating a quantitative difference in the number of phagocytes (Table 1). Whether the increased number of phagocytes in the spleen is due to the recruitment of preexisting or ad hoc differentiated phagocytes from the bone marrow remains to be addressed.

**Table 1:** Summary of quantitative and qualitative data of phagocytes from DC-depleted vs. control mice.

	neutrophils	monocytes
number of cells	>2.8 fold	>3.4 fold
number of Ye^+^ cells	>2.2 fold	>6 fold
Ye phagocytosis/processing	=	=
number of phagocytes with intracellular Ye	>2.2 fold	>6 fold
ROS production	>	=
protection by adoptive transfer	>	n.d.

n.d. not determined.

Ye were more frequently associated with neutrophils (2.2-fold) and monocytes (6-fold) from DC-depleted mice, whereas most of the Ye in control mice were extracellular attached to B cells (Figure 4 and 5). Detailed cellular analysis revealed no qualitative difference in the phagocytosis and processing rates of Ye by neutrophils and monocytes *in vivo* (Figure 5). Calculation of the overall number of intracellular Ye in neutrophils and monocytes revealed 2.8-fold more intracellular Ye in DC-depleted mice compared to control mice. DC-depletion not only increased the number of neutrophils but also enhanced their production of antimicrobial substances (ROS) (Figure 6). Moreover, neutrophils from DC-depleted mice are more efficient in reducing the bacterial load than neutrophils from control mice, indicating that DC depletion enhances the innate anti-bacterial host defense by modulation of phagocyte homeostasis (Table 1). We cannot exclude that other effector mechanism than ROS account for the enhanced bacterial killing capacity of neutrophils. This issue could be assessed by analyzing bacterial killing capacity of neutrophils from CD11c.DOG mice on a gp91phox^−/−^ background.

The increased numbers of phagocytes upon DC depletion were associated with increased serum levels of G-CSF, CXCL1, CCL2, Flt3L, and IL-1α upon DC depletion. G-CSF was shown to induce proliferation of granulocytic precursors and release of mature neutrophils from the bone marrow by downregulation of CXCR4 on their cell surface [32], [33]. CXCL1 was shown to act in cooperation with G-CSF stimulating neutrophil chemotaxis across the bone marrow endothelium [29]. CCL2 mediates the chemotaxis of CCR2^+^ monocytes and macrophages [30]. Systemic infection of mice with *L. monocytogenes* leads to recruitment of CCR2^+^ monocytes via CCL2, into the spleen where they differentiate into TNF- and inducible NO synthase (iNOS)-producing DCs that are essential for control of the infection [34]. CCR2-mediated recruitment of monocytes was also shown to be essential for defense against *Mycobacterium tuberculosis*, *Toxoplasma gondii*, and *Cryptococcus neoformans* infection [35]. It is tempting to speculate that the recruited monocytes in the spleen upon DC depletion as well as upon Ye infection express CCR2, due to the increased serum levels of CCL2.

The specific cellular mechanisms by which DC depletion increases the numbers of phagocytes and promotes enhanced neutrophil responses remain to be determined and are currently under investigation. So far we could show that this regulation is cell contact independent (Figure S6), but mediated by a factor or factors present in the serum upon DC-depletion (Figure 7A and B).

Finally, we could show that DC-depletion prior to infection reduced the bacterial load not only in the case of Ye infection, but also in the case of other bacteria as shown for *S. typhimurium, L. monocytogenes* and *E. coli* infection (Figure 8). Increased ROS production was observed upon infection with *S. typhimurium*, but not with *L. monocytogenes* and *E. coli*, suggesting either other defense mechanisms of activated neutrophils, or the increased number of neutrophils combined with their enhanced ROS production upon DC-depletion is sufficient to protect against these pathogens.

Hochweller et al. recently described for the first time an increased number of neutrophils and monocytes in spleen following DC depletion [25]. Similarly, a previous study showed that bone marrow chimeras of CD11c.DTR and WT mice (another mouse model for conditional DC depletion [36]) develop myeloproliferative disorder (MPD), indicated by massive increase in the number of CD11b^+^ cells after two weeks of DT treatment every second day [37]. Furthermore, constitutive DC depletion in mice also led to MPD at the age of three months [37], suggesting a feedback loop regulating appropriate myelogenesis during homeostasis. In both models, elevated serum levels of Flt3L, a critical factor in the control of DC development [38] and maintenance in the periphery [28], but no changes in M-CSF, GM-CSF, and TNF were observed [37]. Indeed, we also found significantly increased Flt3L in the sera of DC-depleted mice compared to control mice and increased myeloid progenitors in spleen responsive to Flt3L (Figure 2 and [26]), which is likely due to less consumption of Flt3L by DCs in the periphery as mainly immediate DC progenitors and DCs express its receptor Flt3 [39], [40]. Based on our data we favor the notion that, at least in our model, DCs affect the number of neutrophils and monocytes by modulating the production of growth factors (G-CSF and Flt3L) and chemokines (CXCL1 and CCL2) by a yet unknown mechanism.

DC depletion led to increased serum levels of IL-1α, that was recently shown to be produced in response to necrosis and stimulates CXCL1 production by non-immune cells, leading to the attraction of neutrophils [41]. We could exclude necrosis as a side effect of the increased number of phagocytes in the spleen upon DC-depletion using mixed bone marrow chimeras (80% CD11c.DOG/20% C57BL/6, Figure S3). These mice have normal DC numbers (from C57BL/6 bone marrow cells) after 10 days of DT treatment and still 80% of the DCs are depleted due to DT-treatment. If DC death would cause increase in phagocyte numbers, similar numbers of phagocytes should be seen in mixed bone marrow chimeras and CD11c.DOG mice after 10 days of DT treatment. Yet, this was not the case.

Ye infection increased the number of neutrophils in the spleen, and this was more prominent in control mice compared to DC-depleted mice. Additionally, Ye infection increased the levels of G-CSF, CXCL1, and CCL2 in the sera 7–10 times more in control mice compared to DC-depleted mice, indicating that DCs limit neutrophil numbers in the steady state to prevent tissue damage by these cells but are required for their recruitment upon infection. The latter conclusion is supported by recent findings from a bacterial pyelonephritis model that showed, that kidney DCs secrete CXCL2 upon a second instillation with uropathogenic *E. coli* leading to the recruitment of neutrophils and bacterial phagocytosis [42]. The simultaneous DC depletion (CD11c.DTR model) with *E. coli* instillation resulted in markedly delayed recruitment of neutrophils to the kidney, due to less CXCL2 secretion and bacterial clearance [42].

Scumpia et al. showed that DCs are essential in the immune response to sepsis as DC depletion (CD11c.DTR model) reduced the survival of mice in a cecal ligation and puncture (CLP) infection model. Adoptive transfer of BM-DCs improved the survival during this CLP induced polymicrobial sepsis [14], but no changes - in bacterial load or in serum cytokine levels were observed and the underlying mechanism(s) remain unresolved. The differences to our study may be explained by the different mouse model as well as the more severe polymicrobial infection model.

Rapid recruitment of neutrophils and abscess formation is required for bacterial clearance [43]–[45]. Recently it became evident that the bacterial load plays a pivotal role in neutrophil survival [46]. Upon *Staphylococcus aureus* infection the half-life of neutrophils in wound abscesses increased up to 3-fold depending on the inoculum [47]. The increased half-life is presumably mediated by anti-apoptotic signals and cytokines [47]–[49]. In our experimental setting the survival of neutrophils is not influenced by the infection (data not shown).

This study demonstrates for the first time that DC depletion not only increased neutrophil numbers in the spleen but also improved production of ROS and Ye killing capacity. In a burn-injured mouse model, pretreatment of the mice with IL-18 increased neutrophil counts and also enhanced neutrophil phagocytosis, ROS production and killing of methicillin-resistant *S. aureus* [50]. Upon DC depletion no changes in IL-18 serum levels were observed (data not shown), indicating that other factors than IL-18 account for more effective neutrophils in our model. Treatment of burn-injured mice with Flt3L prior to *Pseudomonas aeruginosa* wound infection enhanced neutrophil chemotaxis, bacterial killing and survival [51]. Furthermore, adoptive transfer of neutrophils from Flt3L-treated mice reduced the bacterial load in the spleen, whereas neutrophils from DC-depleted (CD11c.DTR model) and Flt3L-treated mice did not, indicating that Flt3L modifies neutrophil responses via DCs in this model [51]. However, the cellular mechanism remains elusive.

In our study, adoptive transfer of neutrophils from DC-depleted mice into control mice prior to Ye infection reduced the bacterial load in the spleen to the level of DC-depleted mice, whereas adoptive transfer of neutrophils from control mice did not, arguing against altered neutrophil response mediated via Flt3L modified DCs. Thus, neutrophils from DC-depleted mice with enhanced anti-bacterial activity account for this effect. Our results are supported by the finding that enhanced local recruitment of neutrophils in peritonitis-induced sepsis improves bacterial clearance and survival [52].

In conclusion, we provide evidence that DCs differently regulate splenic phagocyte numbers in the steady state or upon bacterial infection. Furthermore, the newly recruited neutrophils upon DC depletion display an improved bacterial killing capacity, thereby accounting for the decreased bacterial load and likely increased survival of these mice upon Ye infection. Beyond the anti-bacterial host defense, these studies point towards a complex interaction between DCs and phagocyte homeostasis by serum factors.

## Materials and Methods

### Mice and infection

Ethics statement: Animal experiments were performed in strict accordance with the German regulations of the Society for Laboratory Animal Science *(GV-SOLAS)* and the European Health Law of the Federation of Laboratory Animal Science Associations (FELASA). The protocol was approved by the Regierungspräsidium Tübingen (Permit Numbers: IM5/08, IZ2/11). All efforts were made to minimize suffering.

Female C57BL/6JolaHsd mice were purchased from Janvier (St Berthevin Cedex, France) and Harlan Winkelmann (Borchen, Germany). CD11c.DOGxC57BL/6 mice [25] were bred under specific pathogen-free conditions in the animal facilities of the University of Tübingen. Mice used for experiments were between 6–9 weeks of age and were provided food and water *ad libitum*.

Mice were infected with the indicated amount of Ye WA-314 (serotype 0∶8), WA-314 expressing GFP [53], *Salmonella enterica* serovar *typhimurium* SL1344, *Escherichia coli* JM83 or *Listeria monocytogenes* ATCC 43251 in 200 µl PBS into the tail vein. As a control, mice were injected only with 200 µl PBS. The bacterial load in the spleen was obtained after plating serial dilutions of the cell suspensions on Müller-Hinton or Luria Bertani agar plates. For systemic DC depletion BAC transgenic CD11c.DOG mice, that express the human diphtheria toxin receptor under control of the CD11c promoter, were injected intraperitoneally or intravenously with 8 ng/g bodyweight of diphtheria toxin (Sigma) in PBS one day prior to Ye infection and daily during infection.

### Cell preparation and culture

Spleens were cut into small pieces and digested for 30 min at 37°C in 2 ml RPMI 1640+2% FBS medium containing collagenase (1 mg/ml; type IV; Sigma-Aldrich) and DNase I (100 µg/ml, Roche). To disrupt DC-T cell complexes, EDTA (0.1 ml, 0.1 M (pH 7.2)) was added and mixing continued for 5 min. Single cell suspensions were made by pipetting the digested organs. Undigested fibrous material was removed by filtration and erythrocytes were lysed with lysis buffer (150 mM NH_4_Cl, 10 mM KHCO_3_, 2 mM NaEDTA). The total number of cells was determined by trypan blue exclusion.

### Flow cytometry & cell sorting

FACS buffer ((PBS containing 1% FBS (Sigma-Aldrich) and 0.09% NaN_3_ (Sigma-Aldrich) was used for all incubations and washing steps. Before staining, cells were incubated for 15 min at 4°C with hybridoma supernatant from 2.4G2 cell line producing anti-FcgRII/III mAb. Cells were stained with anti-CD11c-APC (N418 Miltenyi Biotec), CD11c-PE (N418, eBiosciences), CD8α-PE-Cy7 (53-6.7, eBiosciences), CD4-eFluor450 (RM4-5, eBiosciences), CD19-APC (6D5, Miltenyi Biotec), MHC II-PerCP (M5/114.15.2, Biolegend), Ly6C-PE-Cy7 (HK1.4, Biolegend), Ly6G-FITC (1A8, Biolegend), Ly6G-PE (1A8, Miltenyi Biotec), Gr-1-eFluor450 (RB6-8C5, eBiosciences), CD11b-APC-eFluor780 (M1/70, eBiosciences), CD45.1-eFluor450 (A20, eBiosciences), CD62L-APC (MEL14-H2.100, Miltenyi Biotec) for 20 min at 4°C. To exclude dead cells, 7-aminoactinomycin D (7-AAD; Sigma-Aldrich) or aqua life dead (Invitrogen) was used. Samples were acquired for 6 to 8-colour analysis using a Canto-II flow cytometer (BD Biosciences) with DIVA software (BD Biosciences) and further analyzed using FlowJo 7.5 software (TreeStar Inc). A total of 500,000–1,200,000 cells were acquired.

For the analysis of viable intracellular Ye in splenic phagocytes after intravenous infection of the mice, CD19-expressing cells were depleted from splenic single cell suspensions by MACS technology using CD19 magnetic beads (Miltenyi Biotec) following the manufacturer's protocol. Fc block was performed and cells were stained with Gr-1-FITC (RB6-8C5, BD Biosciences), CD11b-PE (M1/70, BD Biosciences), and CD11c-APC (N418, Miltenyi Biotec) in PBS. DCs, neutrophils, and CD11b^+^CD11c^−^Gr-1^−^ cells were sorted on a FACS Aria cell sorter (BD Biosciences) and reanalyzed on a Canto-II flow cytometer. Cells were treated afterwards with gentamicin (100 µg/ml, Sigma Aldrich) for 30 min at 37°C to kill extracellular bacteria. Cells were then lysed with PBS containing 0.1% tergitol TMN 10 (Sigma-Aldrich) and 0.1% bovine serum albumin (Merck) and the bacterial load in the spleen was obtained after plating serial dilutions of the suspensions on Müller-Hinton agar plates.

### Multispectral imaging flow cytometry (MIFC)

Mice were treated with DT one day prior to infection with 5×10^8^ WA-314 expressing eGFP. After 30 min the spleen was removed and the splenocytes were stained with Ly6C-Pacific blue (HK1.4, Biolegend) and CD11b-APC (M1/70, Biolegend) or CD45R(B220)-Vioblue (RA3-6B2, Miltenyi Biotec) and CD19-APC (6D5, Beckmann Coulter), fixed with 1% paraformaldehyde, permeabilized with 0.1% saponin (Sigma- Aldrich) and 0,5% BSA (Sigma-Aldrich) in PBS and stained with CD107a-PE (1D4B, Biolegend). Images of up to 8,000 Ye-positive events were then acquired with multispectral imaging flow cytometry (MIFC) using an ImageStream equipped with a custom designed 40× objective (0.75 NA) (Amnis corp., Seattle, USA) [54], [55]. Image data were analyzed with IDEAS 3.0 (Amnis corp.), which allows an objective and unbiased analysis of thousands of images per sample on the single cell level. To quantify bacterial uptake regions of interest (mask) were defined for each cell. The first mask covered any fluorescence of the event, independent whether it originated from Ye or from the cells (total event mask). Then a second mask that includes the cytoplasm and nuclei and excludes the plasma membrane was defined (cytoplasm mask). To create this cytoplasm mask, we first created a filled mask based on the lineage markers (e.g. CD19 or Ly6C) that covers the entire cell and excludes the lineage negative Ye on the top of the cell. To make this mask more stringent, it was then eroded by one pixel, i.e. 500 nm. The resulting mask excludes the plasma membrane and specifies the cell interior only. Thereafter, the internalization score was calculated, which is a rescaled ratio of the Ye-GFP intensity in the cytoplasmic mask and in the total event mask. Thus, the higher the score the more Ye were internalized. We counted a cytoplasmic localization of Ye if the internalization score was >2. Thereafter, the subcellular localization of intracellular Ye-GFP was evaluated by calculating the colocalization of Ye-GFP and CD107a using a rescaled Pearson's correlation coefficient, named similarity score [56]. Ye show a high degree of colocalization, if the similarity score is >1.

### Killing assay *in vitro*

Splenocytes were incubated, where indicated, with serum diluted in RPMI +10% FBS (1∶5) for one hour at 37°C. 2×10^6^ splenocytes were incubated with 2×10^6^ Ye for 10 min at 37°C and afterwards serial dilutions were plated on Müller-Hinton agar plates. For kinetic studies (Figure 6A) cells were incubated as described, washed, and incubated further for the indicated time points in RPMI +10% FBS in the presence of gentamicin (100 µg/ml).

### ROS detection

Mice were treated with DT over night and were infected with 5×10^4^ Ye. Mice were sacrificed after 2 h and the spleen was aseptically removed. Spleen cell suspensions were obtained and flow cytometry staining was performed as described above. 3×10^6^ cells were incubated for 20 min at 37°C with 2′, 7′-Dichlorofluorescin diacetate reagent (DCFD, Sigma-Aldrich), washed and analyzed by flow cytometry.

### Adoptive transfer of neutrophils

For the adoptive transfer of neutrophils CD11c.DOG mice or C57BL/6 mice were treated with DT over night (Figure 6D). Splenocytes were obtained from these or from CD45.1 C57BL/6 mice and either B cells depleted using anti-CD19 beads or Ly6G^+^ cells enriched using anti-Ly6G beads and MACS technology as described above. Figure 6D: Flow cytometry staining was performed as follows: Gr-1-FITC, CD11b-APC-Alexa780, and Ly6C-PE-Cy7. Neutrophils were sorted on a FACS Aria cell sorter (BD Biosciences). 1.2 to 1.8×10^6^ neutrophils were adoptively transferred into each C57BL/6 mouse and infected with 5×10^4^ Ye 30 min later. One day post infection CFU per spleen was analyzed by serial dilution. Figure 7C: 4×10^6^ CD45.1^+^ neutrophils were adoptively transferred into each C57BL/6 or DC-depleted CD11c.DOG mouse. 2 h after transfer ROS production by CD45.1^+^Ly6G^+^ cells was analyzed as described above.

### Cytokine detection

Cytokines from sera were analyzed. IFN-γ and FLT3L were measured by ELISA (eBiosciences and R&D, respectively). CXCL1, CCL2, G-CSF, IL-6, and IL12-p40 were measured by multiplex bead technique (Biorad) according to the manufacturer's protocol.

### Immunofluorescence of cryosections

Tissues were embedded in Tissue-Tek OCT compound (Sakura), frozen in liquid nitrogen and stored at −80°C. 5 µm cryostat sections were prepared and dried over night. Slides were fixed for 10 min with ice cold acetone, dried for 1 h and were then rehydrated for 15 min with PBS containing 0.25% bovine serum albumin. After blocking with Fc-block (hybridoma supernatant from 2.4G2 cell line producing anti-FcγRII/III mAb) in PBS containing 10% fetal bovine serum and 5% normal goat serum (Sigma-Aldrich) slides were incubated with polyclonal rabbit anti-yersinia antibodies (WA-v; 5 µg/ml) [57] in PBS containing 10% fetal bovine serum and 5% normal goat serum for 30 min at room temperature. Alexa Fluor 488-labeled goat-anti-rabbit IgG F(ab') fragment (1 µg/ml, Invitrogen) or DyLight 649-labeled goat-anti-rabbit IgG F(ab')_2_ fragment (5 µg/ml, Jackson) were used as secondary antibodies. Slides were blocked with a biotin blocking kit (Vector) and then stained with biotin-conjugated anti-CD11c antibody (HL3, 5 µg/ml, BD Biosciences) or biotin-conjugated anti-Gr-1 antibody (RB6-8C5, 5 µg/ml; eBiosciences) in PBS-10% FBS, washed and then incubated with streptavidin-Alexa Fluor 546 (1 µg/ml in PBS-10% FBS, Invitrogen) for 30 min at room temperature. For triple staining slides were further incubated with FITC-labeled rat-anti-mouse Ly6G antibody (1A8, 5 µg/ml; Biolegend). Nuclei were stained with DAPI (10 µg/ml, Sigma-Aldrich). Slides were mounted in Mowiol (Carl Roth). Labeled cells were visualized with a DMRE fluorescence microscope (Leica) or an Axiovert 200 M fluorescence microscope (Zeiss). Image processing was performed with Adobe Photoshop (Version 8.0.1).

### Mixed bone marrow chimeras

Mixed BM chimera mice were generated as previously described [26] by transferring 2×10^6^ Thy1.2-depleted donor BM cells into 10 Gy-irradiated recipient B6 mice. Donor BM consisted of a mixture of cells from CD11c.DOG CD45.1 mice (DTR^+^) and eGFP mice (DTR^−^) at a ratio of 80∶20. Experiments were started 8 to 10 weeks after reconstitution.

### Statistics

Statistical analysis was performed using the GraphPad Prism 5.0 software (GraphPad, San Diego, CA). Diagrams show mean values ± SD. Statistical analysis was performed using the unpaired two-tailed Student's *t* test. Statistical analysis of survival was performed by using the log-rank test. Data from cytokine production and adoptive transfer experiments were analyzed using one-way ANOVA with Bonferrfoni post test. When data were not normally distributed, a logarithmic transformation was applied prior to the analyses. The differences were considered as statistically significant if p<0.05 (*), p<0.01 (**), p<0.005 (***) or p<0.001 (****).

## Supporting Information

Figure S1 **DC depletion upon DT treatment.** **(A)** Flow cytometry analysis of CD11c^hi^MHC II^+^ cells in the spleen of CD11c.DOG mice (red symbols) mice treated once with DT. The frequency of DCs was assessed at the indicated days post DT treatment. **(B)** DC-depleted (red symbols) and DT-treated control (black symbols) mice were injected i.v. with 5×10^4^ Ye pYV^+^ and daily with diphtheria toxin (DT) starting one day before infection. Bacterial load (CFU) per spleen weight was assessed at the indicated days post infection by plating. Each symbol represents an individual mouse; horizontal lines indicate the mean ± SD. * indicates statistically significant differences (Student's *t*-test). **(C)** Control (black symbols) or CD11c.DOG mice were treated with DT (red symbols) or PBS (grey symbols) 24 h prior to infection with 5×10^4^ Ye pYV^+^. The CFU per spleen were assessed at the indicated days post infection by plating. Each symbol represents an individual mouse; small horizontal lines indicate the mean ± SD. * indicates statistically significant differences. Data were analyzed by one-way ANOVA with Bonferroni post test.
(TIF)

Figure S2 **Gating strategy of monocytes and neutrophils.** Flow cytometry analysis of monocytes (pink gate and cells; Ly6C^hi^CD11b^+^Gr-1^int^Ly6G^−^) and neutrophils (blue gate and cells; Ly6C^+^CD11b^hi^Gr-1^hi^Ly6G^+^) in the spleen from DC-depleted (left) or control mice (right). Numbers adjacent to outlined areas indicate frequency of monocytes and neutrophils.
(TIF)

Figure S3 **Analysis of neutrophils in mixed bone marrow chimeras.** The number of live splenocytes (top panel), the frequency (middle panel) and number (bottom panel) of live neutrophils (SSC^hi^CD11b^+^Gr-1^hi^) in the indicated mice after DT administration. Shown is a representative of three independent experiments (n = 4 mice). **, *p*<0.01; ***, *p*<0.001 (one-way ANOVA with Dunnett post-test).
(TIF)

Figure S4 **Recruitment of monocytes and neutrophils into the spleen upon Ye infection.** **(A–B)** Flow cytometry analysis of monocytes **(A)** and neutrophils **(B)** in the spleen from control (black symbols) and DC-depleted (red symbols) mice treated daily with DT starting one day before infection. Mice were injected with 5×10^4^ Ye pYV^+^ and cells were analyzed at the indicated times. Graphs show the numbers (#) of the indicated cells per spleen. Each symbol represents an individual mouse; horizontal lines indicate the mean ± SD. * indicate statistically significant differences between control DC-non-depleted and DC-depleted mice (Student's *t*-test). Data are representative out of 2 or more independent experiments.
(TIF)

Figure S5 **DC depletion leads to reduced bacterial load 30 min post Ye infection.** Control (black symbols) and DC-depleted mice (red symbols) were injected with 5×10^8^ Ye pYV^+^ and the CFU were analyzed 30 min post infection. Each symbol represents an individual mouse; horizontal lines indicate the mean ± SD. * indicate statistically significant differences (Student's *t*-test). One representative experiment out of 5 is shown.
(TIF)

Figure S6 **Ye are predominantly associated with neutrophils upon DC depletion.** Control (upper panel) and DC-depleted mice (lower panel) were injected with 5×10^8^ Ye pYV^+^ and the spleen was removed 30 min post infection. Immunohistochemical analysis of Ye (blue), DCs (red) and PMNs (green) in spleens, visualized by staining with polyclonal antiserum to Ye and DyLight 649-labeled secondary antibody followed by biotin-labeled monoclonal antibody to CD11c and Alexa Fluor 546-labeled streptavidin and FITC-labeled monoclonal antibody to Ly6G. Original magnification ×20 (top row) and ×40 (bottom row). Arrows indicate DCs colocalized with PMNs. Data are representative out of 2 independent experiments.
(TIF)
