# Supplementary figures and images for: Depletion of Dendritic Cells Enhances Innate Anti-Bacterial Host Defense through Modulation of Phagocyte Homeostasis
Source: PLoS Pathog. 2012 Feb 23;8(2):e1002552. doi: 10.1371/journal.ppat.1002552 (PMC3285606; doi:10.1371/journal.ppat.1002552)

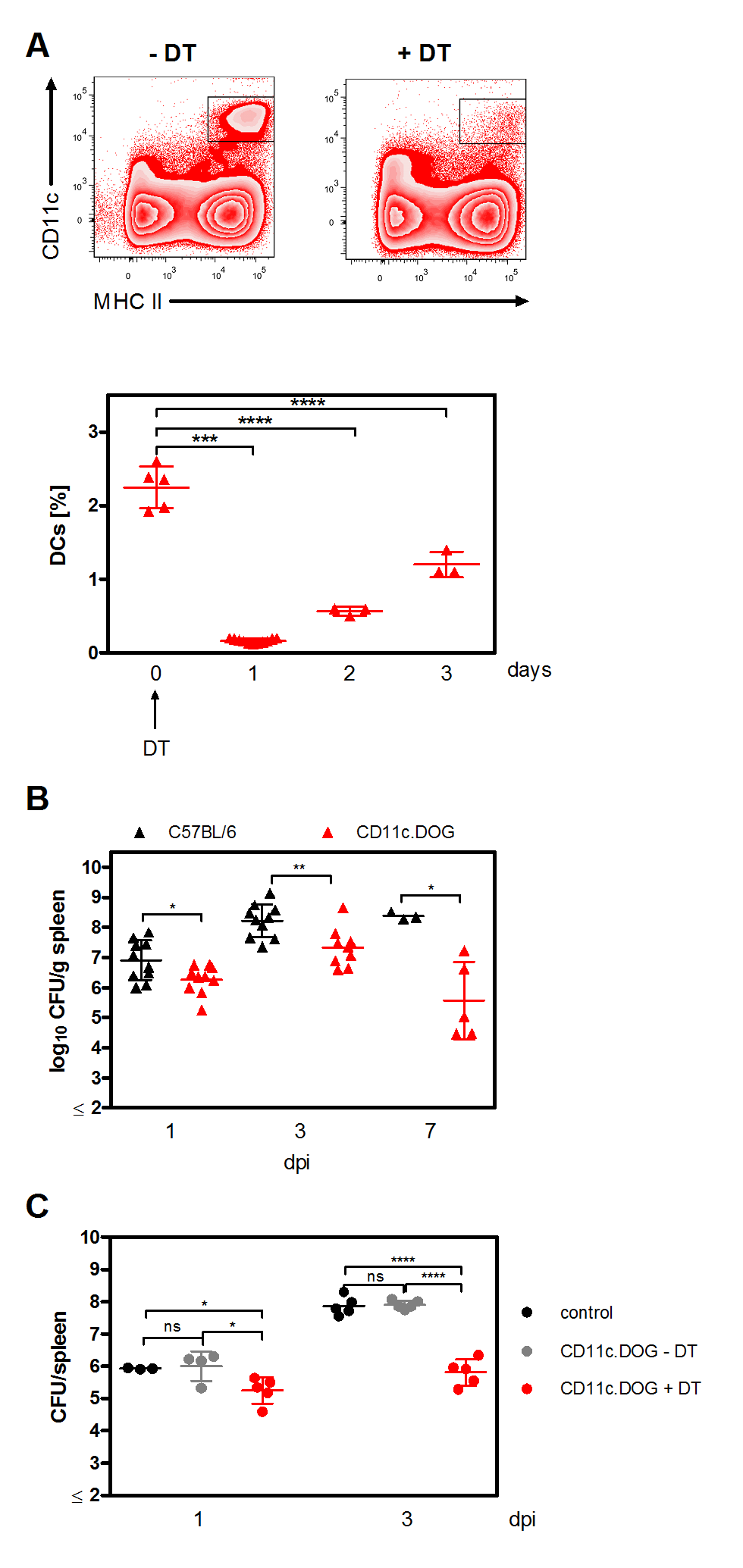

Supplement: Figure S1 — DC depletion upon DT treatment. (A) Flow cytometry analysis of CD11chiMHC II+ cells in the spleen of CD11c.DOG mice (red symbols) mice treated once with DT. The frequency of DCs was assessed at the indicated days post DT treatment. (B) DC-depleted (red symbols) and DT-treated control (black symbols) mice were injected i.v. with 5×10^4^ Ye pYV+ and daily with diphtheria toxin (DT) starting one day before infection. Bacterial load (CFU) per spleen weight was assessed at the indicated days post infection by plating. Each symbol represents an individual mouse; horizontal lines indicate the mean ± SD. * indicates statistically significant differences (Student's t-test). (C) Control (black symbols) or CD11c.DOG mice were treated with DT (red symbols) or PBS (grey symbols) 24 h prior to infection with 5×10^4^ Ye pYV+. The CFU per spleen were assessed at the indicated days post infection by plating. Each symbol represents an individual mouse; small horizontal lines indicate the mean ± SD. * indicates statistically significant differences. Data were analyzed by one-way ANOVA with Bonferroni post test. (TIF) [file ppat.1002552.s001.tif]

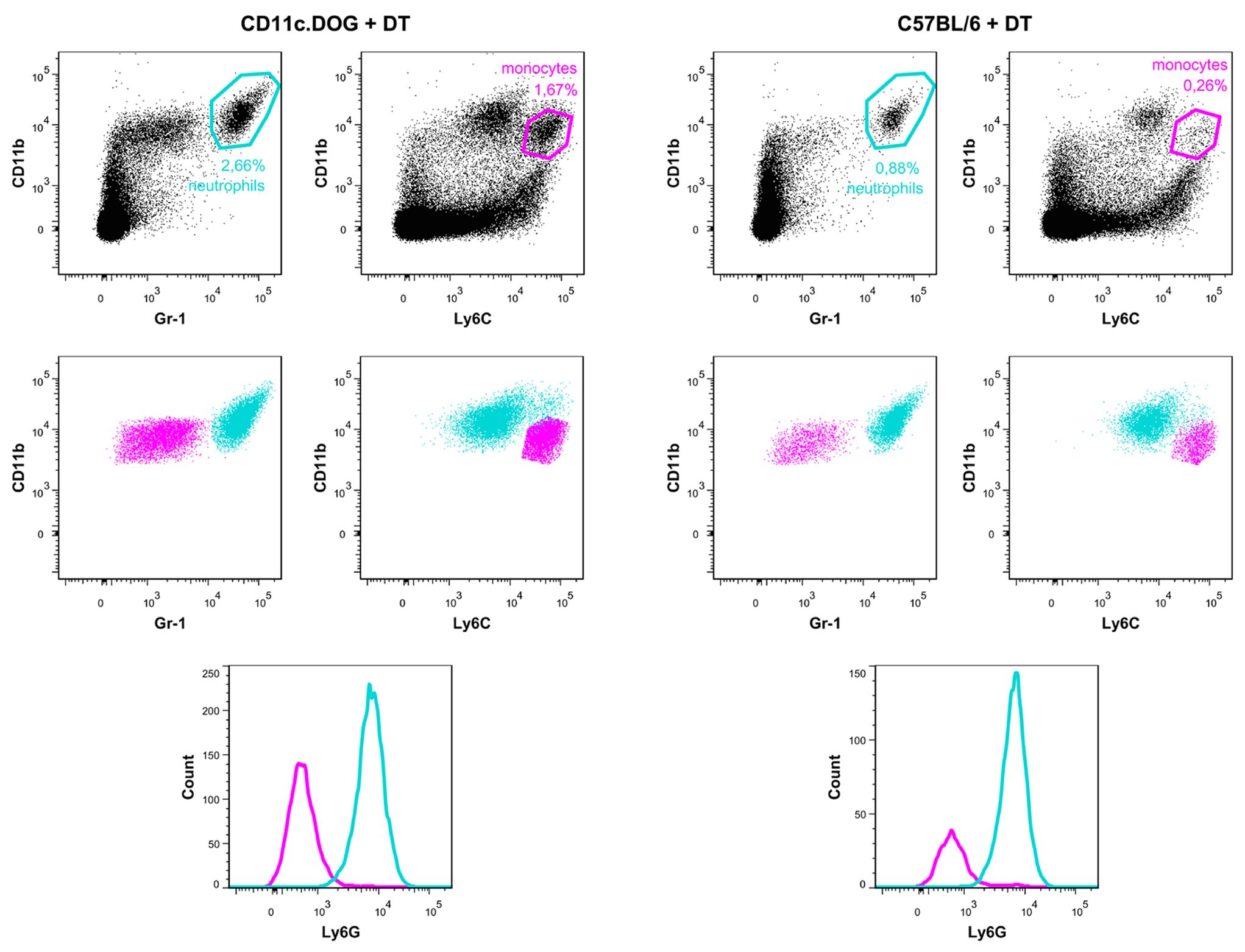

Supplement: Figure S2 — Gating strategy of monocytes and neutrophils. Flow cytometry analysis of monocytes (pink gate and cells; Ly6ChiCD11b+Gr-1intLy6G−) and neutrophils (blue gate and cells; Ly6C+CD11bhiGr-1hiLy6G+) in the spleen from DC-depleted (left) or control mice (right). Numbers adjacent to outlined areas indicate frequency of monocytes and neutrophils. (TIF) [file ppat.1002552.s002.tif]

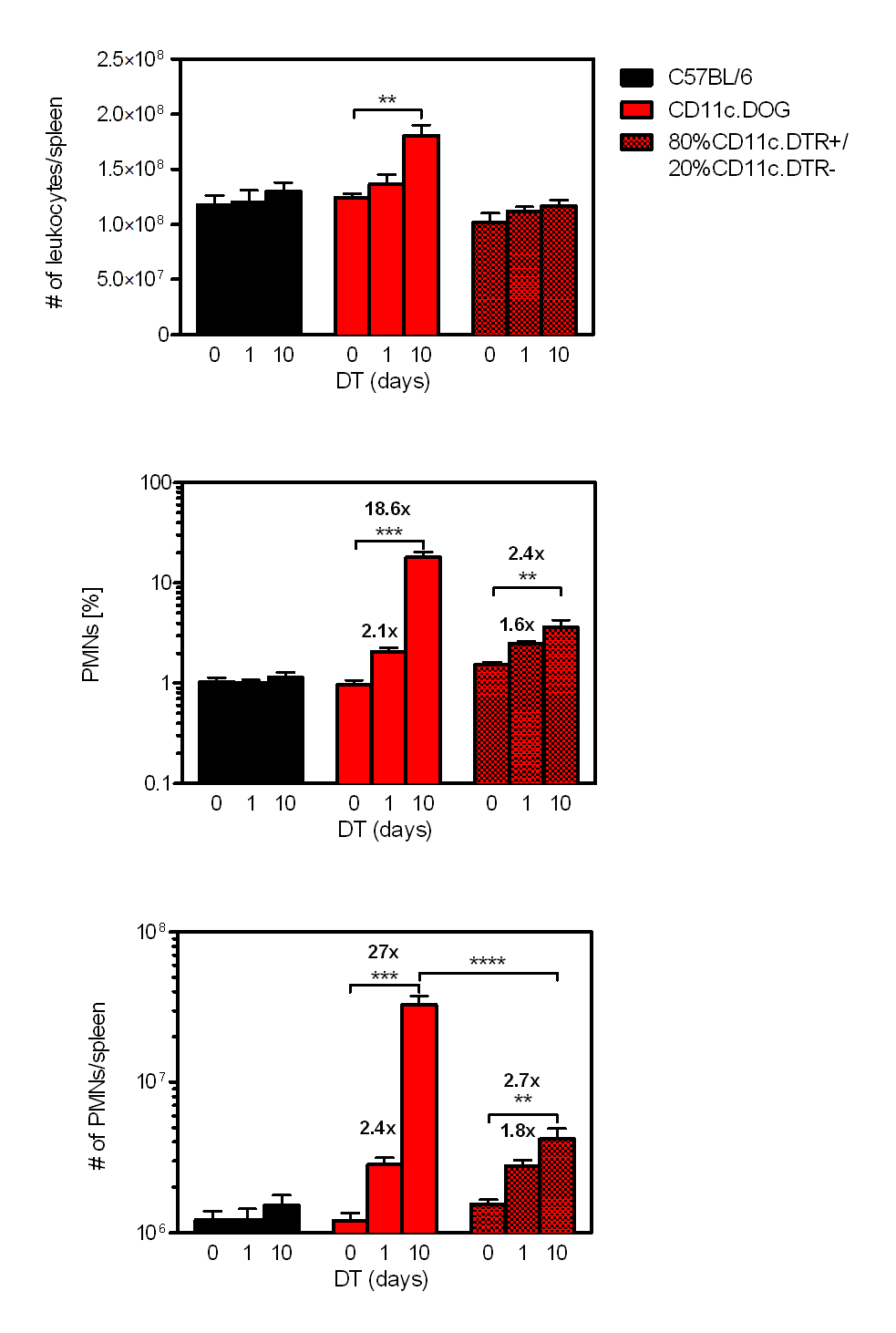

Supplement: Figure S3 — Analysis of neutrophils in mixed bone marrow chimeras. The number of live splenocytes (top panel), the frequency (middle panel) and number (bottom panel) of live neutrophils (SSChiCD11b+Gr-1hi) in the indicated mice after DT administration. Shown is a representative of three independent experiments (n = 4 mice). **, p<0.01; ***, p<0.001 (one-way ANOVA with Dunnett post-test). (TIF) [file ppat.1002552.s003.tif]

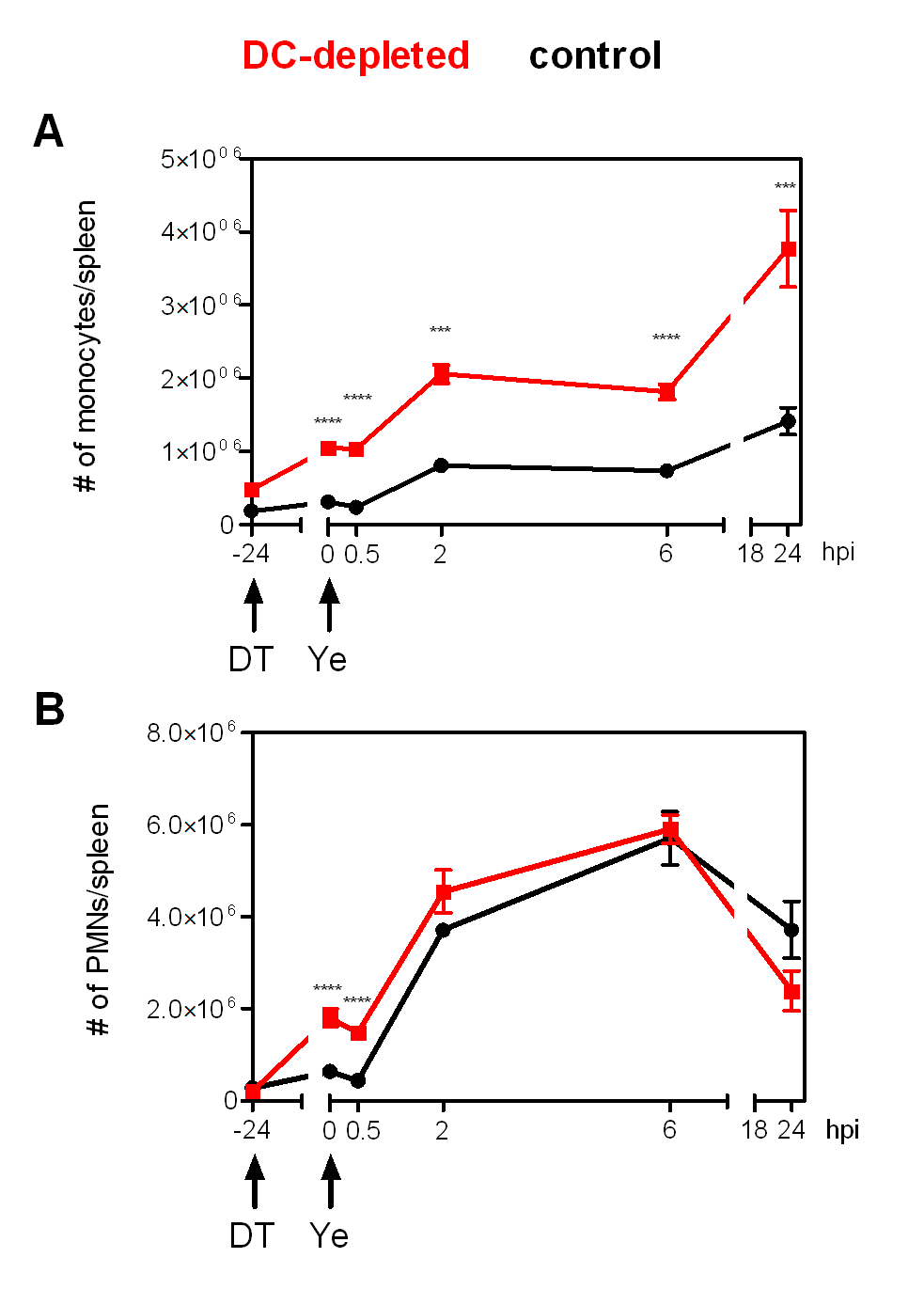

Supplement: Figure S4 — Recruitment of monocytes and neutrophils into the spleen upon Ye infection. (A–B) Flow cytometry analysis of monocytes (A) and neutrophils (B) in the spleen from control (black symbols) and DC-depleted (red symbols) mice treated daily with DT starting one day before infection. Mice were injected with 5×10^4^ Ye pYV+ and cells were analyzed at the indicated times. Graphs show the numbers (#) of the indicated cells per spleen. Each symbol represents an individual mouse; horizontal lines indicate the mean ± SD. * indicate statistically significant differences between control DC-non-depleted and DC-depleted mice (Student's t-test). Data are representative out of 2 or more independent experiments. (TIF) [file ppat.1002552.s004.tif]

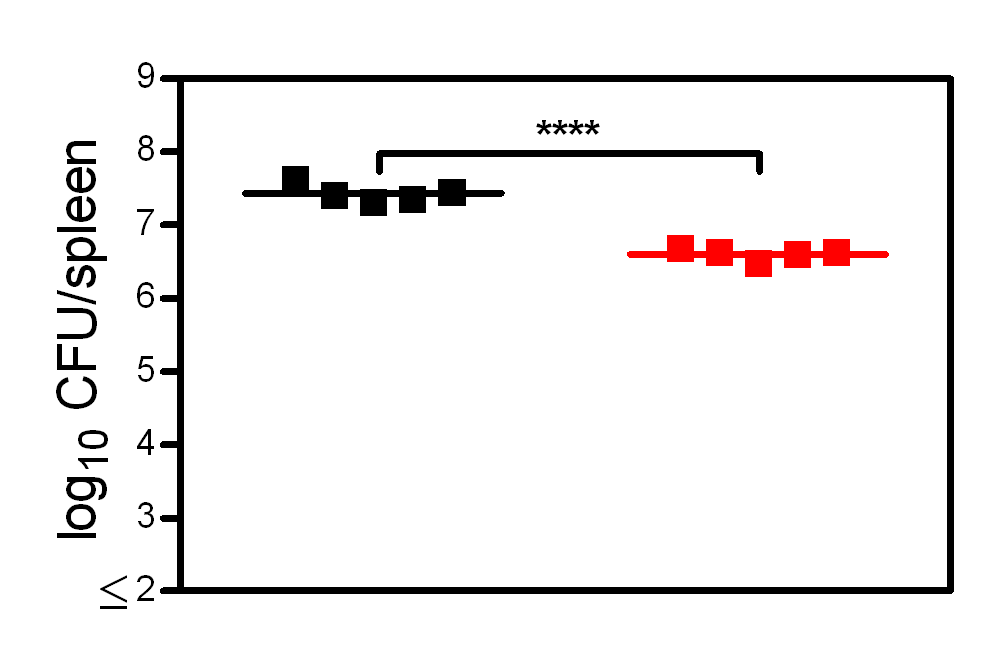

Supplement: Figure S5 — DC depletion leads to reduced bacterial load 30 min post Ye infection. Control (black symbols) and DC-depleted mice (red symbols) were injected with 5×10^8^ Ye pYV+ and the CFU were analyzed 30 min post infection. Each symbol represents an individual mouse; horizontal lines indicate the mean ± SD. * indicate statistically significant differences (Student's t-test). One representative experiment out of 5 is shown. (TIF) [file ppat.1002552.s005.tif]

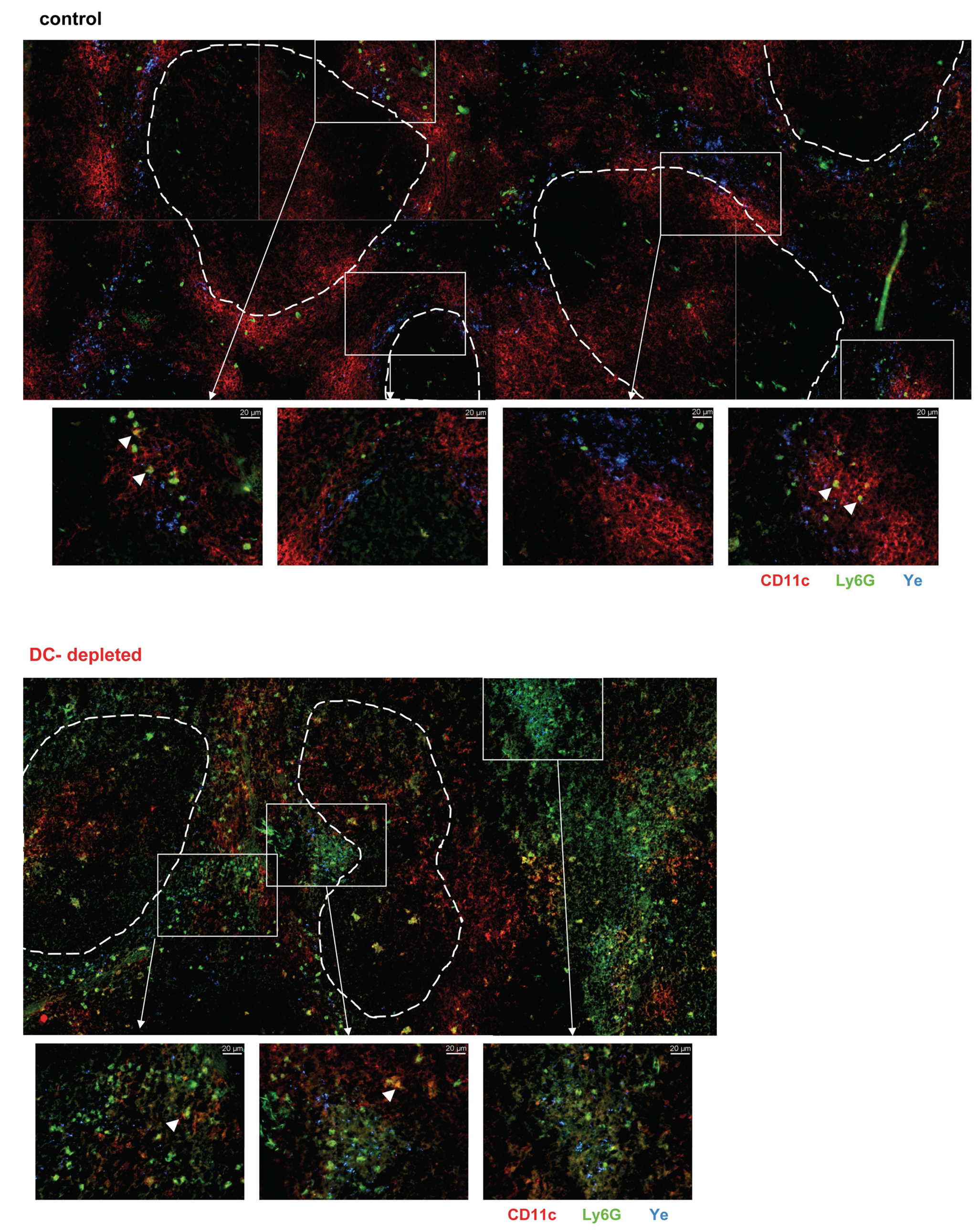

Supplement: Figure S6 — Ye are predominantly associated with neutrophils upon DC depletion. Control (upper panel) and DC-depleted mice (lower panel) were injected with 5×10^8^ Ye pYV+ and the spleen was removed 30 min post infection. Immunohistochemical analysis of Ye (blue), DCs (red) and PMNs (green) in spleens, visualized by staining with polyclonal antiserum to Ye and DyLight 649-labeled secondary antibody followed by biotin-labeled monoclonal antibody to CD11c and Alexa Fluor 546-labeled streptavidin and FITC-labeled monoclonal antibody to Ly6G. Original magnification ×20 (top row) and ×40 (bottom row). Arrows indicate DCs colocalized with PMNs. Data are representative out of 2 independent experiments. (TIF) [file ppat.1002552.s006.tif]
